# Validation of quantitative real-time PCR reference genes for the determination of seasonal and labor-specific gene expression profiles in the head of Western honey bee, *Apis mellifera*

**DOI:** 10.1371/journal.pone.0200369

**Published:** 2018-07-09

**Authors:** KyungHwan Moon, Si Hyeock Lee, Young Ho Kim

**Affiliations:** 1 Department of Applied Biology, College of Ecology & Environmental Science, Kyungpook National University, Sangju, Gyeongbuk, Republic of Korea; 2 Department of Agricultural Biotechnology, Seoul National University, Seoul, Republic of Korea; 3 Research Institute for Agriculture and Life Science, Seoul National University, Seoul, Republic of Korea; 4 Department of Ecological Science, Kyungpook National University, Sangju, Gyeongbuk, Republic of Korea; Philipps-Universitat Marburg Fachbereich Biologie, GERMANY

## Abstract

Honey bee is not only considered an important pollinator in agriculture, but is also widely used as a model insect in biological sciences, thanks to its highly evolved sociality, specialization of labor division, and flexibility of colony management. For an intensive investigation of the seasonal and labor-dependent expression patterns of its genes, accurate quantification of the target gene transcription level is a fundamental step. To date, quantitative real-time PCR (qRT-PCR) has been widely used for rapid quantification of gene transcripts, with reliable reference gene(s) for normalization. To this end, in an attempt to search for reliable reference genes, the amplification efficiencies of six candidate reference genes (*rp49*, *rpL32*, *rpS18*, *tbp*, *tub*, and *gapdh*) were determined. Subsequently, four genes (*rpL32*, *rpS18*, *tbp*, and *gapdh*) with PCR efficiencies of 90% to 110% were evaluated for their expression stabilities with three programs (geNorm, NormFinder, and BestKeeper) and used for normalization of seasonal expression patterns of target genes in the forager and nurse heads. Although the three programs revealed slightly different results, two genes, *rpS18* and *gapdh*, were suggested to be the optimal reference genes for qRT-PCR-based determination of seasonal and labor-specific gene expression profiles. Furthermore, the combined use of these two genes yielded a more accurate normalization, compared with the use of a single gene in the head of honey bee. The validated reference genes can be widely used for quantification of target gene expression in honey bee head although it is still remained to be elucidated the expression levels of the selected reference genes in specific tissues in head.

## Introduction

The Western honey bee, *Apis mellifera* L., is a keystone pollinator in both natural and agricultural ecosystems. In the United States, the monetary value of honey bees as commercial pollinators is estimated at more than $15 billion annually: they pollinate almost 80% of the agricultural fruits and vegetables. Honey bees also produce honey and other products [1]. In addition to its commercial importance, honey bee has been broadly studied as a model insect species because of its highly evolved sociality, specialization of labor division, and flexibility of colony management [2]. The many tasks associated with colony maintenance and growth, such as hive clearing, brood rearing, and foraging, are divided among the worker bees in an age-based fashion, a phenomenon known as polyethism [3, 4]. After emergence from the pupae, individual bees perform the various behavioral tasks in a specific sequence [5]. The youngest bees primarily clean the cells for 3 days, after which they feed and care for larvae and the queen in the central region of the nest for several days (up to 12 days). From days 13 to 20, the middle-age bees engage in hive maintenance and food storage in the peripheral region. At day 20, the bees defend the colony at the entrance of the hive as a guard, and after a few days, they forage for nectar and pollen outside the nest until they die [6, 7].

The population dynamics of honey bee colonies in temperate regions also show a characteristic seasonal pattern. The colony population is composed of several thousand bees in the winter, but it increases up to tens of thousands of adult bees in the summer [8]. According to a recent study [9], in early spring, when the natural brood-rearing activity was not initiated, an artificial supplementation of pollen diet accelerated the induction of brood-rearing activity. Overwintering beehives exhibiting complete cessation of brood rearing resumed brood-rearing activity when they were placed in strawberry greenhouses with a pollen diet. Furthermore, in the foraging season, when highly active beehives were placed in a screen tent, brood-rearing activity was dramatically suppressed; however, brood rearing was restored when the screen tent was removed. These results strongly support the notion that honey bee colonies expeditiously recognize and actively respond to environmental changes. As demonstrated in the previous studies [9–11], seasonal changes in endocrine system status and gene expressions are important factors that allow honey bees to flexibly manage the colonies via passive regulation of their population and labor division.

In this context, identification of the genes involved in regulating colony physiology and their seasonal expression patterns is essential to understand the seasonal changes at the molecular level that allow flexibility in honey bee colony management. As quantitative real-time PCR (qRT-PCR) is a fast, sensitive, replicable, and accurate method that has been widely used for quantification of gene expression level [12, 13], it can be employed to analyze seasonal expression patterns of the genes putatively associated with plasticity in worker bee physiology. To analyze the expression differences of the target genes across different seasons, normalization with reference genes is required to compensate for differences in the amount of RNA content in the collected seasonal honey bee samples. Therefore, reference genes ideally should have a consistent transcription level in both nurse and forager bees over the yearlong cycle, and should not vary in abundance in response to environmental factors [14]. As summarized by Reim et al. (2013), identification and validation of reference genes for qRT-PCR have been carried out in various insect species. In honey bee, reference genes have been investigated in different developmental stages [14], in the brains of workers after a bacterial challenge [15], and in the brains of bees with different ages and social roles [16]. However, the candidate reference genes have not been validated between nurse and forager bees collected over a yearlong cycle. In the present study, in order to find the most suitable references genes for quantification of target gene expression by qRT-PCR in workers with different tasks over different seasons, we collected nurses and foragers over a yearlong cycle on a monthly basis. We then extracted RNA from the head of these bees and validated six candidate reference genes, including three ribosomal proteins (*rp49*, *rpL32*, and *rpS18*), the TATA box binding protein (*tbp*), the tubulin α-1 chain (*tub*), and glyceraldehyde 3-phosphate dehydrogenase (*gapdh*). The suitability of the genes as references was subsequently analyzed using three programs (geNorm, NormFinder, and BestKeeper) for normalized analysis. In addition, after selection of stably expressed reference genes, we validated the normalization effect of the selected reference genes on the expression patterns of *ace2*, which encodes acetylcholinesterase 2 (AChE2).

## Materials and methods

### Insects and sample preparation

The apiary field of in the Seoul National University experiment forest issued the permission for this study. The colonies of *A*. *mellifera* (Italian hybrid) were maintained mainly in the apiary field of in the Seoul National University experiment forest in Gwangju, Gyeonggi-do, Korea (37° 18′ 43.2″N, 127° 18′ 40.8″E) during the foraging season, whereas they overwintered in Goheung, Jeollanam-do, Korea (34° 30′ 38.0″N, 127° 19′ 00.8″E) [9]. From spring to autumn, nurses and foragers were collected according to their ages and behaviors. During winter, however, young bees in the central region and old bees in the peripheral region of the nest were collected as nurses and foragers, respectively, following our previous study [9]. The collected honey bee samples from 10 selected colonies were immediately frozen in dry ice and stored at -70 °C until RNA extraction. Among these 10 colonies stored at -70 °C, three colonies (colonies 6, 8, and 10) were randomly selected and used for this study.

Heads were separated from five foragers and five nurses and completely homogenized with TRI Reagent^®^ (MRC, Cincinnati, OH) with a bullet blender (Bertin Technologies, Montigny-le-Bretonneux, France). Total RNA was extracted with Direct-Zol^™^ RNA MiniPrep Plus (Zymo Research, Irvine, CA) from the heads, and DNase I was treated during RNA extraction procedure. To check for successful degradation of contaminant DNA, following a previous study [14], *rp49* primers were designed to span an intron, which enabled detection of genomic DNA contamination based on amplicon size (442 bp in the case with genomic DNA contamination, in contrast with 150 bp in genomic DNA free condition). Similarly, primers for *rpS32* and *tbp* were designed to amplify 152 and 99 bp, respectively, from cDNA, whereas PCR products of 446 bp and 177 bp are supposed to be detected when there is genomic DNA contamination (S1 Table and Table 1). The RNA concentration and purity were measured in triplicate with a QuickDrop spectrophotometer (Molecular Devices, Sunnyvale, CA). The extracted RNA was stored at -70 °C until use.

**Table 1:** Description, accession number of the reference gene, size and GC percentage of PCR products, qRT-PCR efficiency, and correlation coefficient (R^2^) of the selected reference genes.

Symbol	Accession no.	size (bp)*	GC (%)	Efficiency (%)	R^2^
***rp49***	AF441189	150 (442)	37.6	87.6	0.9980
***rpL32***	XM006564315	181	33.1	105.3	0.9897
***rpS18***	XM625101	152 (446)	35.5	107.6	0.9999
***tbp***	XM623085	99 (177)	34.3	106.8	0.9962
***tub***	XM396338	145	40	82.6	0.9993
***gapdh***	XM393605	188	43.1	95.5	0.9970
***ace2***	NM001040230	139	65.5	100.6	0.9969

* Number in brackets indicate the size of PCR products amplified with genomic DNA

### Primer design and cloning

Six reference genes were selected, based on previous studies with some modification [14–16] (S1 Table). To ensure similar properties for each primer and amplicon of six candidates and *ace2*, the primers were accurately designed with Oligo Calc software (http://biotools.nubic.northwestern.edu/OligoCalc.html) based on the sequence information of selected genes obtained from the NCBI database (https://www.ncbi.nlm.nih.gov/). For subcloning of the candidate reference genes, total RNA extracted from the whole bodies of five randomly selected honey bees was used as a template for the reverse transcription PCR reaction with DiaStar^™^ OneStep RT-PCR kit (SolGent, Daejeon, Korea). The reaction conditions were: 50 °C for 30 min, followed by 15 min at 95 °C (20 sec at 95 °C, 40 sec at 55 °C, 30 sec at 72 °C) × 35 cycles, and 5 min at 72 °C, with gene-specific primers for each gene amplification (S1 Table). PCR products were visualized in 1% agarose gels to ensure amplification specificity, purified with QIAqick PCR purification kit (Qiagen, Valencia, CA, USA), and subcloned into the pGEM^®^-T easy vector (Promega, Madison, MU, USA). The plasmids were transformed into DH5α chemically competent *Escherichia coli* (Zymo Research). Positive clones were identified by colony PCR using M13 forward and M13 reverse universal primer sets. At least three cloned plasmids were sequenced using the M13 universal primers with the ABI PRISM 3730XL Analyzer (Macrogen, Seoul, Korea). After confirmation of the sequences of genes, the positive *E*. *coli* clones were incubated in the LB liquid media at 37 °C for 16 h, and plasmids were isolated with Zyppy^™^ Plasmid Miniprep kit (Zymo Research) and used as PCR templates for calculation of PCR efficiency values.

### Quantitative real-time PCR

All qRT-PCR reactions were performed on the CFX Connect^™^ Real-Time PCR detection system (Bio-Rad, Hercules, CA) with CYBR^®^ GREEN methodology. To determine the PCR efficiency, three points of a 15-fold dilution series of plasmid DNAs (from 1 × 10^−2^ μg) containing each gene as an insert were used to construct the standard curve. Each sample was analyzed in triplicate (technical replicates) in a total reaction volume of 20 μL, containing 5 ρmol of each primer, 10 μL 2× Thunderbird CYBR qPCR Master Mix (Toyobo, Osaka, Japan), and 2 μL of the diluted plasmid. The reactions were performed at 95 °C for 1 min, followed by (95 °C for 15 sec, 55 °C for 15 sec, and 72 °C for 30 sec) × 40 cycles. The specificity of the PCR products was verified by melting curve analysis and gel electrophoresis. Quantification cycle (C_q_) values were determined at the same fluorescence threshold line. C_q_ for each gene was obtained by calculating the arithmetic mean average of triplicates. The PCR efficiency values for each gene were calculated from the given slope after running standard curves by following formula: E = 15^−1/slope^.

To analyze the expression patterns of the selected reference gene, total RNA was extracted from the head samples of three colonies (colonies 6, 8, and 10) collected every month for a year. First strand cDNA was synthesized from 1 μg total RNA with ReverTra Ace^®^ reverse transcriptase (Toyobo), by priming with oligo (dT) in a total reaction volume of 10 μL. For qRT-PCR, each sample was analyzed in duplicate (technical replicate) in a total reaction volume of 20 μL, containing 5 ρmol of each primer, 10 μL 2× Thunderbird CYBR qPCR Master Mix (Toyobo), and 0.5 μL of cDNA. The reactions were performed at 95 °C for 15 min, followed by (95 °C for 15 sec, 55 °C for 15 sec and 72 °C for 30 sec) × 40 cycles. The specificity of the PCR products was verified by melting curve analysis. C_q_ values were determined at the same fluorescent threshold line (0.1) for each gene. The mean and standard error (SE) of C_q_ value were calculated from three different colonies (i.e., three biological replicates), in which the average C_q_ value of each colony was obtained from two technical replicates.

### Reference gene validation

After selecting the stably expressed genes, we chose *ace2*, encoding AChE2, as the target gene to validate the selected reference genes. Honey bee AChE2 has been known to possess high enzymatic activity in the neuronal tissues [17], and the expression levels of this enzyme are significantly higher in nurses than in foragers [7], indicating that neurotransmission, regulated by AChE2, might be involved in olfactory and visual learning and memory. For quantification of relative expression levels of *ace2*, C_q_ values of *ace2* and reference genes (*rps18* and *gapdh*) were obtained from the same sample of each month and then normalized by relative quantification method modified from the original concept of 2^-ΔΔCq^ [18]. When two reference genes were used for normalization, the mean value of their C_q_ values was applied.

### Data analysis

**Fig 1:**
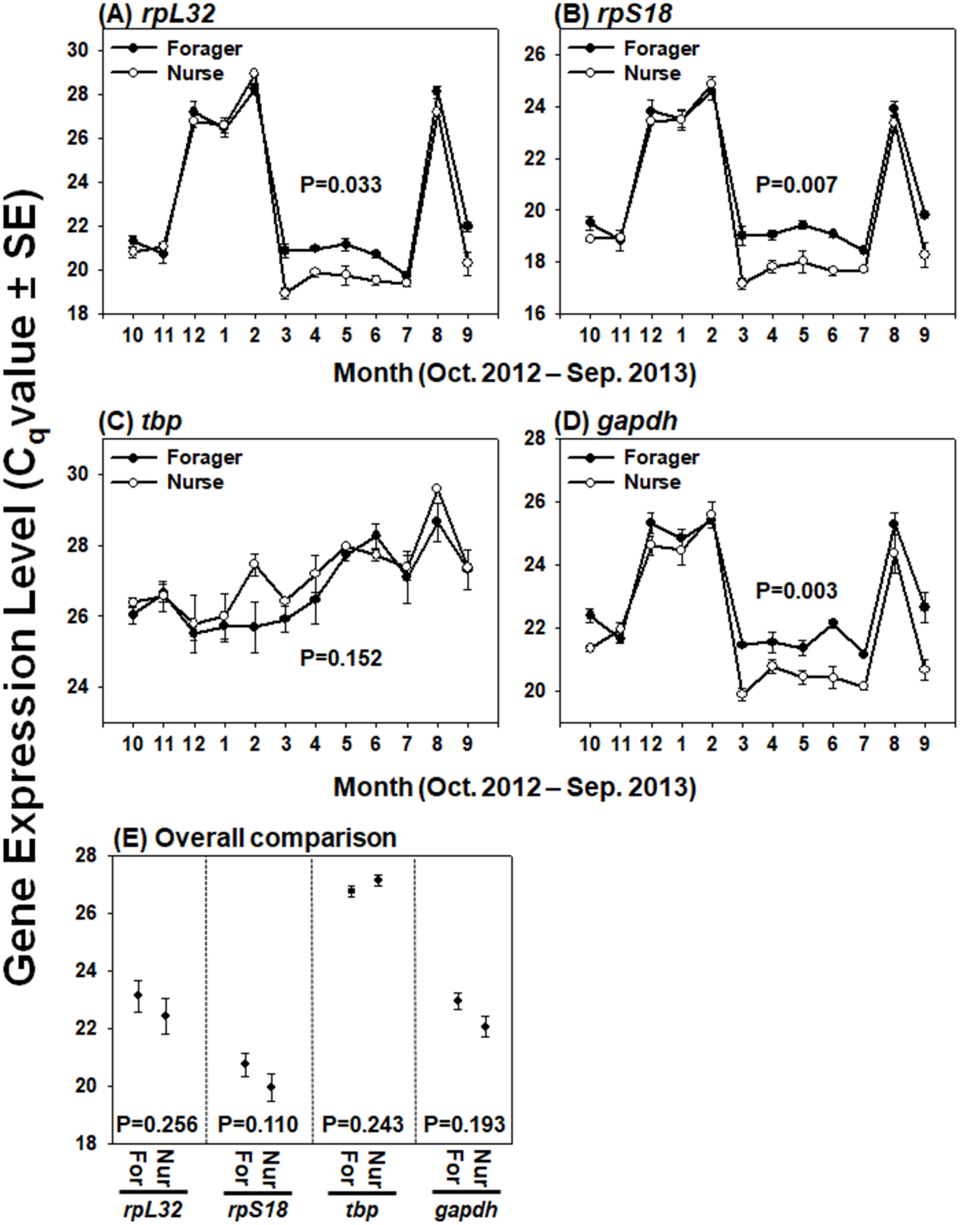
Expression patterns of four candidate reference genes in head samples of foragers and nurses. Seasonal expression patterns, indicated by quantification cycle (C_q_) values of four candidate reference genes over a yearlong cycle, are represented for the foragers and nurses by the closed black circle and open white circle, respectively (A-D). C_q_ values of each gene over the year in foragers and nurses were statistically analyzed with repeated-measures ANOVA (A-D). Average and standard error of C_q_ values of each gene for forager head (For) and nurse head (Nur) were calculated from the data of three colonies for 12 months (E). Integrated expression level of each gene between foragers and nurses were statistically analyzed with Student’s t-test (E).

**Fig 2:**
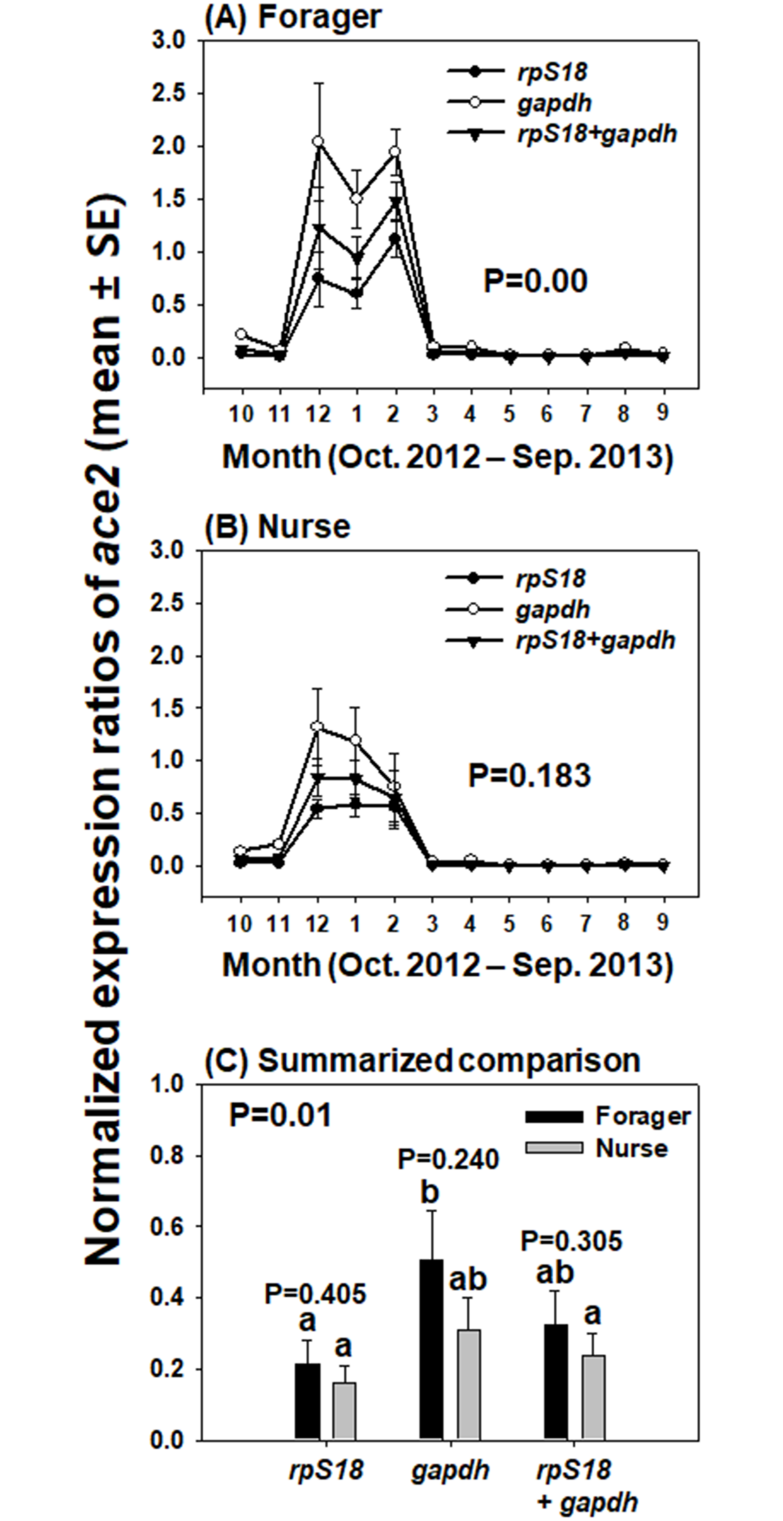
Expression patterns of *ace2* normalized with each of the two reference genes and combination of two genes in forager and nurse heads. The transcript trends of *ace2* in the head samples of foragers (A) and nurses (B) were determined by qRT-PCR using *gapdh*, *rpS18*, and normalization with two reference genes. The expression trends of *ace2* over a yearlong cycle, calculated on a monthly basis across different reference genes, were statistically analyzed (repeated-measures ANOVA; Post-hoc: Tukey’s multiple comparison test, P < 0.05) (A and B). Average and standard error of the expression levels of *ace2*, normalized with a single gene (*rpS18* or *gapdh*) and the combination (two genes), were calculated from the data of three colonies for 12 months (C). P values between forager and nurse sample were analyzed with Student’s t-test, while integrated expression levels of *ace2* for foragers and nurses analyzed with different reference genes (total 6 sample comparison) were compared with one-way ANOVA with Tukey’s multiple comparison test. Different letters indicate significantly different values (P = 0.01).

SPSS for Windows version 23.0 (IBM, Armond, NY, USA) was used for all statistical analyses in this study. Seasonal expression trends of the four candidate reference genes (Fig 1A–1D) and *ace2* as the target gene, normalized with selected reference genes (Fig 2A and 2B), were statistically compared with repeated-measures ANOVA. Between foragers and nurses, integrated expression levels of the four candidate reference genes (Fig 1E) and normalized *ace2* (Fig 2C) were analyzed with Student’s t-test. In addition, average expression level of *ace2* normalized with different reference genes in forager and nurse heads were calculated from the data of three colonies for 12 months, and statistically compared with one-way ANOVA with Tukey’s multiple comparison test (Fig 2C).

**Fig 3:**
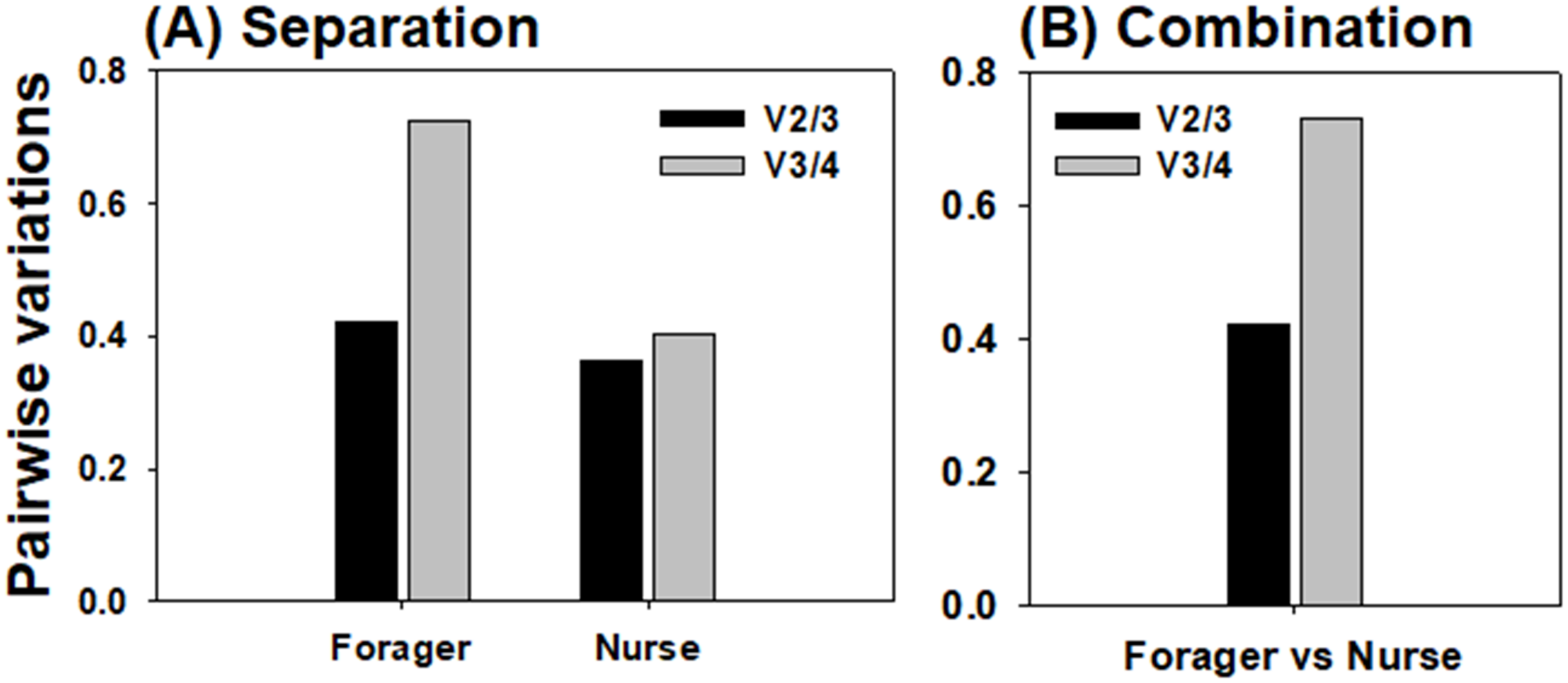
Optimal number of reference genes for normalization calculated by geNorm. Pairwise variations between the normalization factors (NF_n_ and NF_n+1_) were separately analyzed to determine the optimal number of reference genes for normalization in forager head and nurse head samples (A). In order to compare the target gene expression between forager and nurse heads, the optimal number of reference genes for normalization was analyzed using combined data from forager and nurse (B).

For calculation of the candidate gene expression stability, three software programs, geNorm (version 3.1) [19], NormFinder (version 0.953) [20], and BestKeeper (version 1) [21], were used. The geNorm automatically computes an expression stability value (M) for each candidate reference gene, and determines the average pairwise variation (V) of the gene with others to estimate the optimal number of reference genes for accurate normalization. The gene with the lowest M value is the most stably expressed gene in geNorm analysis. NormFinder evaluates the overall variation in the candidate reference gene and calculates its expression stability value, wherein lower values represent more stable genes. BestKeeper determines the geometric mean of the C_q_ values of the genes and standard deviation (SD), therefore lower SD values indicate more stable genes. In addition, it calculates the correlation (R^2^) of each candidate gene with other genes, following which, highly correlated candidate genes are combined to evaluate P values. Therefore, in the BestKeeper analysis, the candidate gene possessing lower SDs, lower P values, and higher R^2^ indicates more stable gene. In this study, in order to find the optimal reference genes, expression stabilities of the candidate reference genes in foragers and nurses were separately analyzed for investigation of target gene expression patterns in different bee workers, and then raw data of qRT-PCR from forager and nurse were combined and gene stabilities were calculated using three programs for a comparative study of target gene expression between foragers and nurses (Tables 2 and 3 and Fig 3).

**Table 2:** Average expression stability values of the five reference genes in forager, nurse, and integration of two tasks calculated by geNorm and NormFinder.

Program	geNorm						NormFinder								
Sample	Forager		Nurse		Forager + Nurse ^a^		Forager			Nurse			Forager + Nurse ^a^		
Ranking	gene	Average M value	gene	Average M value	gene	Average M value	Gene	Stability value	Standard error	Gene	Stability value	Standard error	Gene	Stability value	Standard error
**1**	*rpS18*	0.843	*rpS18*	0.866	*rpS18*	0.84	*gapdh*	0.021	0.027	*gapdh*	0.025	0.03	*gapdh*	0.022	0.019
**2**	*gapdh*	1.017	*gapdh*	1.072	*gapdh*	1.021	*rpS18*	0.062	0.02	*rpS18*	0.072	0.025	*rpS18*	0.067	0.014
**3**	*rpL32*	1.103	*rpL32*	1.142	*rpL32*	1.104	*tbp*	0.077	0.021	*tbp*	0.085	0.023	*tbp*	0.082	0.015
**4**	*tbp*	2	*tbp*	2.107	*tbp*	2.021	*rpL32*	0.096	0.024	*rpL32*	0.1	0.022	*rpL32*	0.096	0.017

^a^ Raw data from forager and nurse were combined and expression stabilities of each gene was calculated using geNorm and NormFinder.

**Table 3:** Gene expression stability values of the five reference genes in forager, nurse, and integration of two tasks calculated by BestKeeper.

	Forager					Nurse					Forager + Nurse^d^				
Ranking	Gene	GM (C_q_) ^a^	SD ^b^	CD (R^2^) ^c^	P value	Gene	GM (Cq)	SD	CD (R2)	P value	Gene	GM (Cq)	SD	CD (R2)	P value
**1**	***tbp***	30.49	1.53	0.978	0.001	***tbp***	27.13	0.77	0.301	0.341	***tbp***	26.93	0.86	0.192	0.368
**2**	***rpS18***	21.44	2.71	0.999	0.001	***gapdh***	21.97	1.8	0.937	0.001	***gapdh***	22.42	1.67	0.93	0.001
**3**	***gapdh***	23.71	2.73	0.981	0.001	***rpS18***	19.78	2.55	0.956	0.001	***rpS18***	20.2	2.34	0.944	0.001
**4**	***rpL32***	23.38	3.7	0.991	0.001	***rpL32***	22.16	3.29	0.965	0.001	***rpL32***	22.54	3.11	0.952	0.001

^a^ SM refers to the geometric mean of C_q_ value

^b^ SD indicates the standard deviation of C_q_ value

^c^ CD means Coefficient of determination value

^d^ Raw data from foragers and nurses were combined, and the expression stabilities of each genes were calculated with BestKeeper.

## Results

### Amplification specificity and efficiency

Prior to conducting the qRT-PCR, we confirmed the specificity of amplification. Six potential reference genes were initially amplified by reverse transcription PCR with designed primer sets (S1 Table), with total RNA extracted from the head of honey bees as a template, and the amplicons were visualized on the 2% agarose gel. All amplicons showed a single band (S1 Fig). Gene-specific amplification of the genes was also confirmed by a single peak in real-time melting temperature analysis. In particular, PCR products amplified with primer sets of *rpL32*, *rpS18*, and *tbp* designed to determine the genomic DNA contamination were visualized as a single band on the agarose gel and a single peak in melting curve analysis, indicating complete genomic DNA digestion after DNase I treatment. In the PCR efficiency calculation, five candidate reference genes displayed high linear regression coefficients that were superior to 0.99 except *rpL32* (0.9897; Table 1). The PCR amplification efficiency values obtained from the qRT-PCR of the candidate genes ranged from 82.6% to 107.6% (Table 1), and we selected four reference genes (*rpL32*, *rpS18*, *tbp*, and *gapdh*) with PCR efficiencies ranging from 90% to 110% for further reference gene validation in this study.

### Expression patterns of reference genes

Expression patterns of the four candidate reference genes in the head samples of foragers and nurses were investigated over a yearlong cycle. Across the different samples and different seasons, the four tested genes showed variable C_q_ values, ranging between 17.15±0.373 (*rpS18* in nurse in Mar) and 29.58±0.026 (*tbp* in nurse in Aug) (Fig 1). Interestingly, the expression levels of *rpL32*, *rpS18*, and *gapdh* were relatively lower in winter (Dec-Jan) and hot summer season (Aug) in both foragers and nurses (Fig 1). In the case of *tbp*, however, the fluctuation in expression pattern was relatively low during a year (Fig 1C). Although all genes exhibited similar seasonal expression trends between foragers and nurses (Fig 1A–1D), *rpL32* (P = 0.033) (Fig 1A), *rpS18* (P = 0.007) (Fig 1B) and *gapdh* (P = 0.003) (Fig 1D) showed statistically different expression levels between two workers. In contrast, similar C_q_ values of *tbp* (P = 0.152) (Fig 1C) between forager and nurse head samples were observed. When the average expression levels of the four genes in heads calculated from C_q_ values over the 12 months were compared between foragers and nurses, the overall transcription levels were not statistically different for any of the genes (P > 0.05) (Fig 1E).

### Seasonal expression stability of reference genes

The average expression stability values (M values) for each reference gene were calculated by the geNorm program. As previous studies suggested that M value under 1.5 (M < 1.5) is an acceptable criterion for selection of qRT-PCR reference genes [13], three candidate genes with M values under 1.5 in all analysis (forager, nurse, and forager+nurse) were indicated as reliable reference genes for the monitoring of seasonal expression patterns of the target gene (Table 2). Among them, *rpS18* was the most stably expressed in foragers (M = 0.843), nurses (M = 0.866), and the combination of two workers (M = 0.840), followed by *gapdh* and *rpL32*, which ranked as the second and third most stable genes, respectively. On the other hand, *tbp* exhibited the most unstable expression across all samples (Table 2). In addition, the optimal numbers of reference genes for accurate normalization were calculated by geNorm (Fig 3). Pairwise variation analysis revealed that V_2/3_ was lower than V_3/4_ in both foragers and nurses (Fig 3A). In the integration data from forager and nurse, V_2/3_ was also lower than V_3/4_ (Fig 3B). Based on the average M values of the four candidates (Table 2), therefore, the combination of *rpS18* and *gapdh* is considered as the reference gene of choice for normalization, in order to determine target gene expression patterns in two different bee tasks or in comparisons between foragers and nurses over a yearlong cycle by qRT-PCR.

According to the NormFinder analysis, *gapdh* showed the most stable expression gene, whereas *tbp* was the least stable gene across all samples (Table 2). The stability rank from the most stable (lowest stability value) to the least stable (highest stability value) gene was: *gapdh > rpS18* > *tbp* > *rpL32* in all samples (Table 2).

Based on the standard deviation (SD) of the C_q_ values, the stability values for the expression of four candidate reference genes were calculated using BestKeeper. The most stable genes were identified according to the lowest SD. The BestKeeper analysis revealed that *tbp* was the most stable gene in forager head (SD = 1.53), in nurse head (0.77), and for the two task combination (0.86) (Table 3). As judged by SD, *rpL32* was the least stable gene in all samples. However, in the cross-comparison, when the BestKeeper index was calculated from the pairwise correlation between genes, *tbp* was not significantly correlated with other genes, as indicated by the relatively low coefficient of determination (R^2^) and high P value (P > 0.001) (Table 3). In the forager head, four candidates (*tbp*, *rpS18*, *gapdh*, and *rpL32*) were significantly correlated to the BestKeeper index, calculated as the geometric mean of the C_q_ values of the other genes, whereas three genes (*gapdh*, *rpS18*, and *rpL32*) showed significant correlation to the BestKeeper index in nurse head (R^2^ > 0.95 and P ≤ 0.001) (Table 3). In the integrated analysis of foragers and nurses, furthermore, *rpL32* demonstrated the highest coefficient of determination (R^2^>0.95) and the lowest P value (P ≤ 0.001), while *gapdh* and *rpS18* also showed relatively high CD values (R^2^ > 0.93) and high P values (P ≤ 0.001) (Table 3). These results suggested that three of the genes (*gapdh*, *rpS18*, and *rpL32*) are significantly correlated with each other, and two genes (*gapdh* and *rpS18*) among them were also ranked as the most stably expressed gene in geNorm and NormFinder analyses (Table 2).

### Validation of reference genes

For investigation of the seasonal and labor-dependent expression patterns of target genes in honey bee head, *gapdh* was suggested to be the most stable gene as a qRT-PCR reference gene, according to the analysis with NormFinder (Table 2). In addition, geNorm analysis revealed that combination of two reference genes might be necessary for accurate normalization (Fig 3), and that two genes (*rpS18* and *gapdh*) are most suitable for the determination of both, the seasonal expression patterns in the head (regardless of labor division) and the labor-dependent (i.e., nurse vs. forager) expression patterns (Table 2). Therefore, a single (*rpS18* or *gapdh*) or combined use of these two genes for normalization was validated by comparing the seasonal expression patterns of *ace2* as the target gene (Fig 2). In forager head samples, expression of *ace2* normalized with a single gene (*rpS18* or *gapdh*), and a combination of these two genes resulted in similar seasonal expression trends, but statistically different seasonal levels (P = 0.00) (Fig 2A), indicating that selection of reference gene can affect the quantification of target gene expression level in forager head. In contrast, no significantly different seasonal transcription patterns and *ace2* levels in nurse were observed between different normalization methods (Fig 2B). In the comparative analysis of the average *ace2* expression levels between the forager and nurse heads during 12 months, the *ace2* expression level normalized with *gapdh* in forager head was significantly higher than those calculated with *rpS18* in forager and nurse, and with combination of the two genes in nurse head (P < 0.05) (Fig 2C). In the comparison of *ace2* transcription levels normalized with the same reference gene between foragers and nurses, however, no significant differences were obtained (P > 0.05) (Fig 2C). The validation of selected reference gene indicates that the analysis of target gene expression can be affected by different reference genes, and normalization with two reference genes may give more reliable results compared with a single gene.

## Discussion

Honey bee is a well-known social insect that shows age-dependent labor division, and is a good model insect to study molecular physiology in brain with respect to social development in insects. In addition, the physiological status of honey bee can be different between foraging and overwintering season, during which induction and reduction of colony social activity are observed, respectively. In this study, therefore, we investigate the transcript levels of four candidate reference genes and evaluate their expression stability using three different algorithms (geNorm, NormFinder, and Best Keeper), in the head of foragers and nurses collected over a yearlong cycle on a monthly basis. We find the most optimal reference genes and compare them between forager and nurse, prior to investigating the seasonal expression trends of a target gene in two groups of honey bees.

All the tested genes displayed fluctuating C_q_ values over different seasons. All the genes are considered housekeeping genes, as they are involved in either protein expression (*rpL32*, *rpS18*, and *tbp*) [22, 23] or metabolism, including glycolysis (*gapdh*) [24]. Therefore, this finding suggests that the physiological status of honey bee head can be considerably affected by seasonal factors. In fact, the overall expression levels of these genes were relatively lower in winter and summer (rainy season), when honey bee colony activity is usually reduced compared with the main foraging season [8–11]. In addition, brood-rearing activities are significantly reduced in winter [8] and hypopharyngeal glands produce high protein levels for brood rearing [25], explaining high C_q_ values of of *rpL32*, *rpS18* and *gapdh* in head samples containing hypopharyngeal glands in broodless season. In contrast, metabolic activity of winter honey bee has been suggested to remain high for keeping long-term memory [26] or endothermic heat production by shivering thermogenesis [27] in winter. Therefore, the reason of different C_q_ values of *rpL32*, *rpS18* and *gapdh* in between overwintering and foraging season should be elucidated. These wide variations of C_q_ values for the genes also result in high SD values in the BestKeeper analysis (Table 3). In addition, seasonal expression levels of *rpL32*, *rpS18*, and *gapdh* were significantly different between foragers and nurses (P < 0.05) (Fig 1). In the previous study, protein production rates in nurse bees were significantly higher than those in foragers in hypopharyngeal glands [25]. Considering that head samples contain hypopharyngeal glands in this study, higher C_q_ values of *rpL32*, *rpS18* and *gapdh* observed in forager than in nurse (see the period of March–July in Fig 1A, 1B and 1D) can be affected by their different expression levels between forager and nurse in hypopharyngeal glands foraging seasons. Although Cq values of *tbp* were not significantly different between nurse and forager bees (P = 0.152) unlike other reference genes, the lowest expression stability of *tbp* precluded its use as a reference gene. When the average C_q_ values of each gene were compared from forgers and nurses, however, every gene exhibited statistically similar C_q_ values throughout the year for foragers and nurses (Fig 1E).

Although the C_q_ values of the candidates varied with the season and different tasks in the present study, in accord with other studies [14–16], we also measured the expression stabilities of the genes and find the most appropriate gene for qRT-PCR using three programs (geNorm, NormFiner, and BestKeeper). According to geNorm analysis, three genes (*rpS18*, *gapdh*, and *rpL32*) demonstrated average M values under 1.5, indicating their suitability as reference genes (Table 2), and selection of two most stable genes was suggested to be optimal for accurate normalization (Fig 3A). These two genes, *rpS18* and *gapdh*, were also ranked as the second and first most stable genes, respectively, by NormFinder analysis (Table 2). Furthermore, although SD values for *rpS18* and *gapdh* were approximately 2.7, they exhibited high coefficients of determination (R^2^ > 0.98) and low P values (P = 0.001), as indicated by the BestKeeper index, thereby showing a significant correlation with other genes (Table 3). When the monthly collected data over the year were used for comparing the target gene, *rpS18* and *gapdh* were consistently identified as the most stable genes according to geNorm and NormFinder (Table 2), and the normalization factor analysis suggested that a combination of these two genes would improve the output (Fig 3B). However, BestKeeper analysis still revealed that *gapdh* and *rpS18* generally possess high CDs and low P values (Table 3). It can be interpreted that, because BestKeeper validates gene stability based on C_q_ values and SD values, *gapdh* and *rpsS18* were likely not ranked as highly stable genes due to their widely fluctuating expression levels during a year (Fig 1B and 1D). Although, three programs, geNorm, NormFinder, and Best Keeper, revealed slightly different sensitivity toward co-regulated reference genes [28], *rpS18* and *gapdh* are consequentially strongly suggested to be suitable reference genes for a reliable determination of the seasonal and labor-dependent (nurse vs. forager) target gene expression patterns for a yearlong cycle, but these two genes are still suggested to be used for the reference genes with the careful consideration because of their fluctuating expression levels during a year (Fig 1B and 1D). These two genes have also been previously found as suitable reference genes in honey bees. Reim et al. (2013) revealed that *gapdh* was consistently expressed in honey bee adult brain from day 0 to 12 after emergence and in pollen and nectar collectors. In addition, *rpS18* and *gapdh* have been identified as optimal reference genes in the honey bee head in the context of bacterial infection [15]. When a single gene (*rpS18* or *gapdh*) and the combination of these two genes were compared for the estimation of *ace2* expression levels, the ace2 transcript quantification was significantly affected by the gene that was chosen as the reference gene in foragers, but not in nurses (Fig 2A and 2B), indicating that the target gene expression level should be calculated by normalization with two genes (*rpS18* and *gapdh*), at least in foragers.

In conclusion, we validated two reference genes for qRT-PCR analysis of honey bee head. Although it is still remain to be elucidated the expression stabilities of the reference genes in specific tissues in head including brain, hypopharyngeal glands and other tissues, based on the results obtained from three programs, *rpS18* and *gapdh* were determined to be most suitable as reference genes, and the combination of these two genes were suggested be the best method for accurate normalization for studies on the overall expression trends of the target gene over a yearlong cycle and comparison between foragers and nurses in whole head tissue of honey bee.

## Supporting information

S1 Table Sequence information, size, GC percentage, and melting temperature of primers for qRT-PCR assay. (DOCX)

S1 Fig PCR amplification of candidate reference genes. Six potential reference genes were amplified by reverse transcription PCR from total RNA extracted from head of honey bee. Each amplicon was visualized on 2% agarose gel.
(TIF)
