# Supplementary material for: Validation of quantitative real-time PCR reference genes for the determination of seasonal and labor-specific gene expression profiles in the head of Western honey bee, Apis mellifera
Source: PLoS One. 2018 Jul 9;13(7):e0200369. doi: 10.1371/journal.pone.0200369 (PMC6037379; doi:10.1371/journal.pone.0200369)
Supplement: S1 Table — (DOCX) [file pone.0200369.s001.docx]

**S1 Table. Sequence information, size, GC percentage, and melting temperature of primers for qRT-PCR assay**

| Symbol | Full gene name | Accession no. | Primers (5'->3') | | Size (bp) | GC (%) | Tm (℃) | reference |
| --- | --- | --- | --- | --- | --- | --- | --- | --- |
| *rp49* | Ribosomal protein 49 | AF441189 | Forward | CGTCATATGTTGCCAACTGGT | 21 | 48 | 59.5 | [14] |
|  |  |  | Reverse | TTGAGCACGTTCAACAATGG | 20 | 45 | 56.4 |  |
| *rpL32* | Ribosomal protein L32 | XM006564315 | Forward | AGTAAATTAAAGAGAAACTGGCGTAAA | 27 | 30 | 60.8 | [16] with modification |
|  |  |  | Reverse | TTAAAACTTCCAGTTCCTTGACATTAT | 27 | 30 | 60.8 |  |
| *rpS18* | Ribosomal protein S18 | XM625101 | Forward | GATTCCCGATTGGTTTTTGAATAG | 24 | 38 | 60.3 | [15] with modification |
|  |  |  | Reverse | AACCCCAATAATGACGCAAACC | 22 | 45 | 60.1 |  |
| *tbp* | TATA-box-binding protein | XM623085 | Forward | TGGCAGCAAGAAAGTATGCTAG | 22 | 45 | 60.1 | [15] with modification |
|  |  |  | Reverse | TCACATCACAGCTGCCTACC | 20 | 55 | 60.5 |  |
| *tub* | Tubulin alpha-1 chain | XM396338 | Forward | CGAGCATTCAGATTGCGCTTTT | 22 | 45 | 60.1 | [15] with modification |
|  |  |  | Reverse | TCGCAACGACGCTGTTATTGAA | 22 | 45 | 60.1 |  |
| *gapdh* | Glyceraldehyde 3-phosphate dehydrogenase | XM393605 | Forward | CACCTTCTGCAAAATTATGGCG | 22 | 45 | 60.1 | [16] with modification |
|  |  |  | Reverse | ACCTTTGCCAAGTCTAACTGTTAA | 24 | 38 | 60.3 |  |
| *ace2* | Acetylcholinesterase 2 | NM001040230 | Forward | TCCGGAGTTGATCACGATATTC | 22 | 45 | 60.1 | This study |
|  |  |  | Reverse | CATGTAACTCCAGGGAGCGT | 20 | 55 | 60.5 |  |

|  |  |
| --- | --- |
