# Supplementary figures and images for: Validation of quantitative real-time PCR reference genes for the determination of seasonal and labor-specific gene expression profiles in the head of Western honey bee, Apis mellifera
Source: PLoS One. 2018 Jul 9;13(7):e0200369. doi: 10.1371/journal.pone.0200369 (PMC6037379; doi:10.1371/journal.pone.0200369)

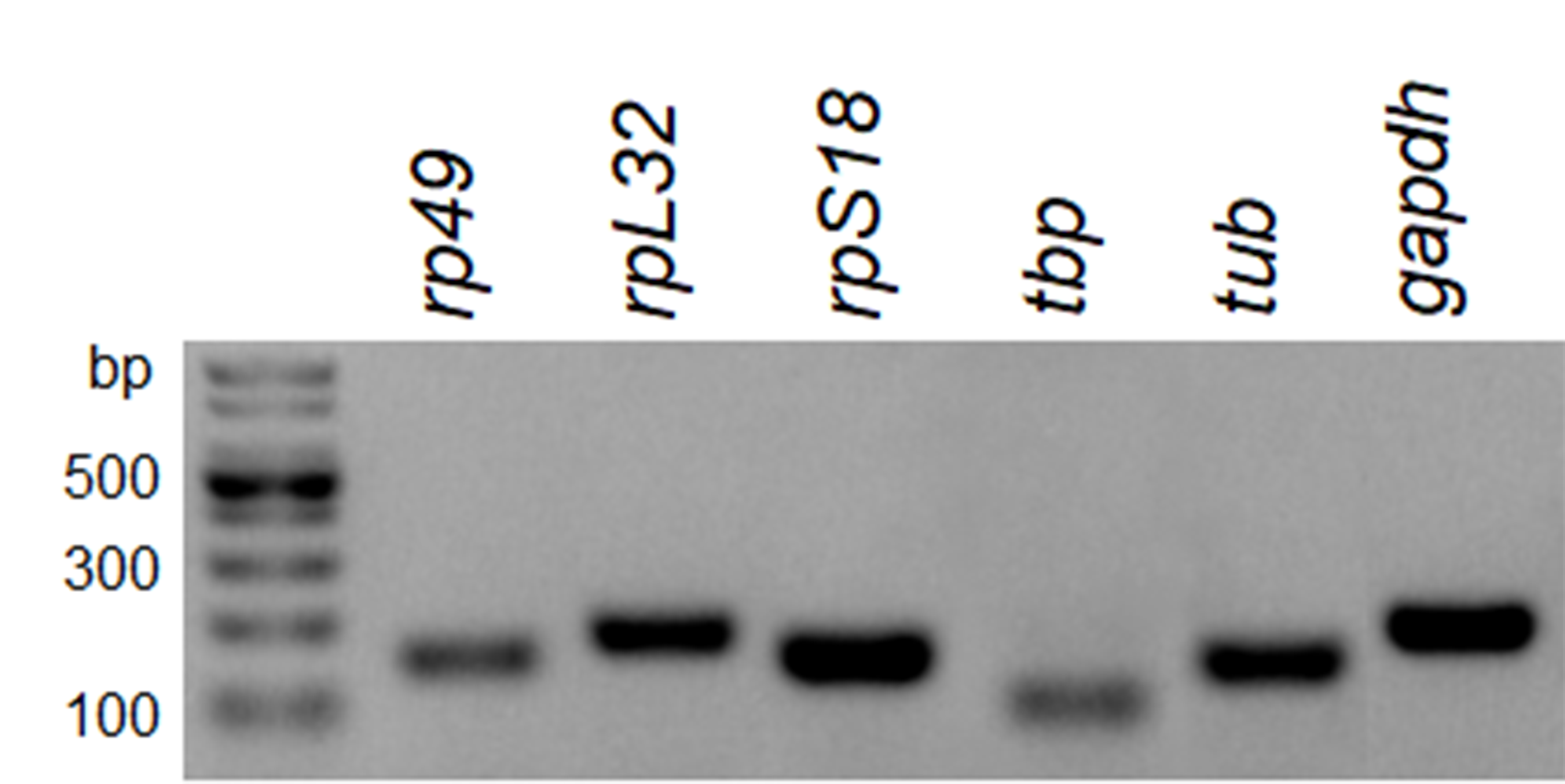

Supplement: S1 Fig — Six potential reference genes were amplified by reverse transcription PCR from total RNA extracted from head of honey bee. Each amplicon was visualized on 2% agarose gel. (TIF) [file pone.0200369.s002.tif]
